# Use of ultrasonography in the evaluation of urinary retention in critically ill patients *

**DOI:** 10.1590/1518-8345.6618.4026

**Published:** 2023-10-09

**Authors:** Karina Rodrigues Lopes, Beatriz Maria Jorge, Maria Helena Barbosa, Elizabeth Barichello, Adriana Cristina Nicolussi

**Affiliations:** 1 Hospital de Clínicas da Universidade Federal de Uberlândia, Uberlândia, MG, Brasil.; 2 Universidade Federal de Mato Grosso do Sul, Três Lagoas, MS, Brasil.; 3 Universidade Federal do Triângulo Mineiro, Departamento de Enfermagem na Assistência Hospitalar, Uberaba, MG, Brasil.

**Keywords:** Intensive Care Units, Physical Examination, Urinary Retention, Ultrasonography, Critical Care Nursing, Urinary Catheterization, Unidades de Cuidados Intensivos, Examen Físico, Retención Urinaria, Ultrasonografía, Enfermería de Cuidados Críticos, Cateterismo Urinario, Unidades de Terapia Intensiva, Exame Físico, Retenção Urinária, Ultrassonografia, Enfermagem de Cuidados Críticos, Cateterismo Urinário

## Abstract

**Objective:**

to measure urinary volume through bladder ultrasound, performed by a nurse in critically ill patients, after removal of the indwelling urinary catheter and to verify the related factors on urinary retention.

**Method:**

quantitative, observational and cross-sectional study, carried out with 37 critically ill patients of both sexes, over 18 years of age, with removal of indwelling urinary catheter in the last 48 hours. A questionnaire containing sociodemographic and clinical variables and an ultrasound examination were used. Data were presented through frequency distribution, centrality and variability measures, association using Fisher`s exact test and, for analysis multiple binomial logistic regression analysis.

**Results:**

the 37 patients were mostly male, with a mean age of 54.9 years. The measurement of urinary volume by ultrasound ranged from 332.3 to 950 ml, and 40.54% of patients had urinary retention. Urinary retention was significantly associated with the occurrence of urinary tract infection, intestinal constipation and spontaneous overflow diuresis. Patients with urinary tract infection were 7.4 times more likely to have urinary retention.

**Conclusion:**

bladder ultrasonography was effective in measuring urinary volume after removal of the indwelling urinary catheter and and may contribute to the detection of urinary retention.

## Abstract (es)

**Objetivo:**

medir el volumen urinario por medio de la ecografía vesical, realizado por una enfermera en pacientes críticos, después de la retirada de la sonda urinaria permanente y verificar los factores relacionados en la retención urinaria.

**Método:**

estudio cuantitativo, observacional y transversal, realizado con 37 pacientes críticos de ambos sexos, mayores de 18 años, con retiro de catéter vesical permanente en las últimas 48 horas. Se utilizó un cuestionario con variables sociodemográficas y clínicas y el examen ecográfico. Se utilizó un cuestionario con variables sociodemográficas y clínicas y el examen ecográfico. Los datos fueron presentados a través de distribución de frecuencias, medidas de centralidad y variabilidad, asociación mediante la prueba exacta de Fisher y, para el análisis, regresión logística binomial múltiple.

**Resultados:**

de los 37 pacientes, en su mayoría fue de sexo masculino, con una edad média de 54,9 años. La medición del volumen urinario por ecografía osciló entre 332,3 y 950 ml, y el 40,54% de los pacientes presentó retención urinaria. La retención urinaria se asoció significativamente a la aparición de infección urinaria, estreñimiento intestinal y diuresis por rebosamiento espontáneo. Los pacientes con infección del tracto urinario tenían 7,4 veces más probabilidades de tener retención urinaria.

**Conclusión:**

la ecografía vesical fue eficaz para medir el volumen urinario después de retirar el catéter urinario permanente y puede contribuir a la detección de retención urinaria.

## Abstract (pt)

**Objetivo:**

mensurar o volume urinário por meio da ultrassonografia de bexiga, realizada por enfermeiro em pacientes críticos, após a remoção do cateter vesical de demora, e verificar os fatores relacionados na retenção urinária.

**Método:**

estudo quantitativo, observacional e transversal, realizado com 37 pacientes críticos de ambos os sexos, idade superior a 18 anos, com retirada de cateter vesical de demora nas últimas 48 horas. Foram utilizados um questionário contendo as variáveis sociodemográficas e clinicas e o exame de ultrassonografia. Os dados foram apresentados por meio da distribuição de frequência, medidas de centralidade e de variabilidade, associação pelo teste exato de Fisher e, para análise a regressão logística binomial múltipla.

**Resultados:**

dos 37 pacientes, a maioria era do sexo masculino, com média de idade de 54,9 anos. A mensuração do volume urinário pela ultrassonografia variou de 332,3 a 950 ml, sendo que 40,54% dos pacientes apresentaram retenção urinária. A retenção urinaria apresentou associação significativa para a ocorrência de infecção do trato urinário, constipação intestinal e diurese espontânea por transbordamento. Pacientes com infecção urinária tiveram 7,4 vezes mais chance de apresentar retenção urinária.

**Conclusão:**

ultrassonografia de bexiga foi eficaz para mensurar o volume urinário após a remoção do cateter vesical de demora e poderá contribuir na detecção da retenção urinária.

**Highlights:**

**(1)** Ultrasonography of the bladder showed an advantage for a better nursing diagnosis.

**(2)** Critical patients had urinary retention after removal of urinary catheter.

**(3)** Overflow incontinence was detected after removal of the urinary catheter.

**(4)** Patients with urinary tract infection were 7.4 times more likely to have retention.

## Introduction

The Indwelling urinary catheter (IUC) is a device widely used within the Intensive Care Unit (ICU) during the care of critically ill patients, and it is essential to implement actions such as early removal of the device to prevent catheter-associated urinary tract infection (CAUTI) ^(^ 1 ^-^ 2 ^)^. 

However, the removal of the IUC can generate a risk of Urinary Retention (UR), which is the incomplete emptying of the bladder ^(^ 3 ^-^ 4 ^)^, because during treatment and hospitalization in the ICU, bodily changes arise in patients, such as: generalized anasarca, injuries, altered level of consciousness, presence of invasive devices, dressings and problems that become obstacles to the correct nursing diagnosis for UR by the nurse ^(^ 5 ^)^. 

Risk factors for UR in patients under critical care are often associated with the use of drugs, length of stay of the indwelling bladder device, restriction of the patient to the bed, age and Urinary Tract Infection (UTI) ^(^ 2 ^-^ 3 ^)^. 

UR can trigger tachycardia, pain, psychomotor agitation, UTI, bladder muscle damage, overflow incontinence, renal failure and pyelonephritis ^(^ 6 ^)^. 

The usual treatment for UR is to perform intermittent bladder catheterization, a procedure performed by nurses, which, even with risks such as infections, urethral trauma, stenosis, bleeding and pain, is preferable to IUC ^(^ 3 ^)^. 

It is plausible to withdraw the IUC for the prevention of infections, but on the other hand, it is important that the nurse has detailed skills to use the nursing diagnosis to prevent the risk of UR and UR in this population, since both cause harm to care ^(^ 7 ^)^. 

Recognizing the related factors the manifestation of UR is essential for nursing care for critical patients, with the aim of preventing complications and providing better quality nursing care. Inspection, percussion and palpation, for the implementation of the UR nursing diagnosis, have limitations due to the low semiological specificity of the physical examination ^(^ 8 ^)^, compared to that obtained with imaging techniques such as bladder ultrasonography (US) ^(^ 1 ^,^ 3 ^,^ 9 ^)^. 

The use of bladder US at the bedside for UR nursing diagnosis contributes to nurses’ decision-making and patient safety, preventing complications and unnecessary procedures.

Nurses are supported by legislation, in the Resolution of the Federal Nursing Council - COFEN 679/2021, which determines that nurses can handle US equipment, if trained to use it, the application of reports and diagnoses being prohibited ^(^ 10 ^)^. 

US can be considered an extension of the physical examination for the nurse, and in this case it has the purpose of measuring the urinary volume, so that the professional can adopt the best approach for each case ^(^ 10 ^)^. 

An integrative review evaluated five studies and listed the main advantages of using US as: effectiveness in measuring urinary volume, reducing the unnecessary use of IUC and the risk of UTI, in addition to early detection of UR ^(^ 11 ^)^. 

Seeking to propose new nursing care strategies, this study aimed to measure the urinary volume through bladder ultrasound, performed by a nurse in critically ill patients, after removal of the indwelling urinary catheter, and to verify the related factors in urinary retention.

## Method

### Study design

This is a quantitative, observational cross-sectional study, guided by the Standards for Quality Improvement Reporting Excellence (SQUIRE) tool.

### Setting

The study was carried out in a highly complex teaching hospital in the city of Uberlândia, MG, Brazil. The hospital has 30 adult ICU beds for the treatment of critically ill patients.

### Population

Surgical, neurological and clinical patients aged 18 years or older, admitted to the ICU, in need of support for organ dysfunctions, intensive monitoring and assistance by a multidisciplinary team.

### Selection and sampling criteria

Adult and older adult patients of both genders who used IUC, which had been removed in the previous 48 hours, were included. The following were excluded: patients in the postoperative period of urological surgeries and kidney transplants; pregnant women; patients with bladder trauma; bladder cancer; peritoniostomies; with ascites and patients with chronic renal failure; with hemodynamic instability and with noradrenaline infusion greater than 50 ml/h; brain death; and palliative or end-of-life care.

To calculate the sample size, an UR prevalence of 27% was used ^(^ 12 ^)^, the number of beds in the ICU (30) and the average hospitalization/month of 91 admissions, reaching a sample of 272 patients, for a period of six months (June to November/2020). However, due to the negative impacts of the pandemic, such as: delay in authorizing the start of data collection on site, lack of family visits to obtain the Free and Informed Consent Form from the legal representative of sedated (unconscious) patients, authorization of only one researcher for data collection and new restrictions imposed in March/2021, it was not possible to reach the estimated sample. Therefore, non-probabilistic convenience sampling was performed ^(^ 13 ^)^, that is, 52 eligible patients were included during the data collection period, of which one refused, eight died and six were discharged, ending the sample with 37 subjects. 

### Data collection instruments and study variables

An instrument containing sociodemographic and clinical variables related to UR was used, submitted to content validation by three expert judges in the thematic area, selected by the Lattes Platform, with a minimum PhD degree and contacted by email. The apparent content validation was performed according to the experts` knowledge to verify the clarity, understanding and presentation of the instrument. After analysis, the instrument was considered valid with 90% agreement among the judges.

The variables sociodemographic and clinical addressed in the questionnaire were: age, gender, medical specialty, neurological evaluation, presence of comorbidities, use of anesthetics and opioid analgesics, presence of UTI, presence of constipation, length of stay of the IUC and the measurement of urinary volume after device removal.

Neurological assessment was performed using the Glasgow Coma Scale (GCS). It is used to assess the level of consciousness and the severity of brain damage and/or permanent sequelae. It is composed of three main components: eye opening, verbal response and motor response. Each component is evaluated, and points are assigned, which are added to obtain a score ranging from three to 15 points, where the lowest value indicates a high degree of neurological involvement ^(^ 14 ^)^. 

The Richmond Agitation-Sedation Scale (RASS) was applied to patients under mechanical ventilation, with sedation or sedoanalgesia. It is used to assess the degree of agitation and sedation, with scores ranging from the aggressive, violent and dangerous patient (+4), going through various stages, to the extreme, which is the inability to be awakened, not responding to the sound of the voice or to the stimulus (-5). Its use provides better assistance to patients in the ICU, as it avoids the administration of excessive sedation, reduces the time of mechanical ventilation and hospital stay ^(^ 15 ^)^. 

### Assessment protocol and nursing diagnosis

For the implementation of the physical examination of nursing using US of the bladder, the protocol “Clinical evaluation for the nursing diagnosis of urinary retention in adult patients” validated in Brazil was applied ^(^ 16 ^)^, which adopts the clinical and urine volume parameters considered for UR greater than or equal to 400 ml. This protocol was adhered to due to the congruence that the document presents with the population included in this study. 

To measure urinary volume, the Logiq V2 ultrasound equipment (GE Healthcare, Milwaukee, Wisconsin, 2018) was used, with a convex transducer with a bandwidth of 1.6 to 4.6 MHz, while involuntary urinary elimination was measure by weighing disposable diapers using a BP Baby digital scale (Filizola), both available in the ward.

Each patient was evaluated only once, the US of the bladder was performed within a minimum interval of four and a maximum of 48 hours after removal of the IUC.

Both the theoretical model used to conceptualize the UR diagnosis and the nursing interventions followed the definition of the International Classification for Nursing Practice (ICNP), created in 1989 by the International Council of Nurses (ICN). In the ICNP ^®^ catalog, each diagnosis is composed of a conceptual core and a minimum set of characteristics that delimit the concept itself and constitute what are called clinical indicators (signs and symptoms) that are part of the definition of the diagnosis that allows the nurse to decide whether to establish it or not. For this study, the construct Urinary Elimination for adult patients undergoing neurorehabilitation was used, validated in Brazil ^(^ 17 ^)^. 

### Data collection procedures

First, a pilot test was carried out with nine patients to determine the applicability of the instruments and the evaluation protocol. The final sample consisted of 37 subjects evaluated from December 2020 to February 2021, as the inclusion of new patients was interrupted due to a new pandemic peak with restrictions described above.

Sociodemographic and clinical data were collected from the patient`s electronic medical record. The anamnesis, the physical examination, the evaluation by means of ultrasound and establishment of the nursing diagnoses were carried out by the nurse and assistant researcher in the adult ICU, in the morning, afternoon and night shifts, after having been duly qualified in a course to assess the volume bladder with the use of auxiliary resources – point of care.

### Data treatment and analysis

The collected data were entered into an Excel ^®^ spreadsheet, through double entry for validation, and subsequently imported into the statistical program Statistic Package for the Social Sciences (SPSS) version 23.0 for Windows. 

Categorical variables were analyzed according to descriptive statistics with absolute and relative frequency distribution, while for quantitative variables were used measures of centrality (central, mean and median) and variability (amplitude and standard deviation). A statistical significance level of 5% was considered, that is, α = 0.05.

Fisher’s exact test was performed to detect possible associations of the variables considered predictors (age group, gender, use of sedatives, opioids, presence of UTI, presence of constipation and spontaneous diuresis due to overflow or not) for the occurrence of UR. The multiple binomial logistic regression analysis was adjusted for gender, age group (adult and older adult) and presence of previous UTI, with UR as the outcome, considering p<0.05 as statistically significant.

### Ethical aspects

The research was approved by the Research Ethics Committee (REC) of the proposing and co-participating institutions, with the number of opinions: 3,952,840 and 4,050,074, respectively. All participants and/or legal representatives signed the Free and Informed Consent Form.

## Results

A total of 37 patients participated in the study, predominantly male, 23 (62.16%); the average age was 54.9 years, with a minimum of 22 years and a maximum of 87 years; 18 (48.65%) patients were hospitalized for neurology, 14 (37.84%) for surgery and five (13.51%) for clinical care.

On assessment, 16 (43.24%) patients were classified as sedated/drowsy and 14 (37.84%) as alert and calm according to the RASS. As for the neurological pattern, using the GCS, an average of 10 points was obtained, with a minimum of three and a maximum of 15 (SD = 4). The most used opioid and sedatives were respectively: Methadone (10 - 27.3%), Midazolam (26 - 70.27%) and Fentanyl (24 - 64.86%).

The most common comorbidities were arterial hypertension (12 - 32.43%) and diabetes mellitus (11 - 29.73%). During hospitalization, constipation and UTI were found in 32 (86.49%) and 12 (32.43%) patients, respectively.

After removal of the IUC, there was an interval of 20.5 hours on average for conducting the physical examination and performing the US of the bladder. The evaluated patients had a maximum of four procedures of relief urinary catheterization (RUC), performed within 24 hours after removal of the IUC. Table 1 presents the results regarding the use of the IUC and the measurement of urinary volume. 

**Table 1 -:** Distribution of patients according to duration of Indwelling urinary catheter (IUC) use, evaluation time after IUC removal, relief urinary catheterization (RUC) interval, number of RUC performed, involuntary loss of urine and urinary measurement by ultrasonography of the bladder (n=37). Uberlândia, MG, Brazil, 2020-2021

**Variable**	**N**	**Mean**	**Standard deviation**	**Minimum**	**Maximum**	P25*	**Median P50** ^†^	**P75** ^‡^
IUC ^§^ usage time (days)	37	11.86	7.99	2	31	5	10	17
Evaluation time after IUC ^§^ removal (hours)	37	20.54	14.64	4	48	6	24	24
RUC ^||^ Range (hours)	14	9.08	14.66	0	72	0	6	8
RUC ^||^ number performed in the last 24 hours	14	0.92	1.32	0	4	0	0	2
Involuntary urinary loss	12	387.73	223.91	100	700	200	300	582.5
Urinary volume measured by US ^¶^	37	332.3	230.73	10	950	160	292	500

^*^P25 = First quartile (25%) of values; ^†^P50 = Second quartile (50%) of values; ^‡^P75 = Third quartile (75%) of values; ^§^IUC = Indwelling urinary catheter; ^||^RUC = Relief urinary catheter; ^¶^US = Ultrasound

Twelve patients presented involuntary urinary elimination in a diaper, when examined by the nurse; among these, nine had their residual urinary volume (post-voiding) greater than 400 ml measured by US of the bladder, confirming the occurrence of urination due to overflow and the presence of UR; another six subjects were diagnosed with UR after the US, totaling 15 patients (40.54%).

Through the application of the ICNP Catalog, in addition to the UR nursing diagnosis, another three were found in the studied population: overflow incontinence, impaired urination and risk of UR. The interventions listed in that catalog were compatible with those carried out, the main ones being: evaluating characteristics of urinary elimination, evaluating bladder distention, monitoring bladder volume with ultrasound, performing bladder catheterization under aseptic technique, changing diapers every three hours or whenever needed.

As shown in Table 2, the incidence of UR was higher for adults (less than 60 years old), men, with the presence of UTI, intestinal constipation and who had spontaneous diuresis (overflow or not), being statistically significant (p<0.05) for the last three. Associations were also made with the use of sedatives and opioids, but without statistical significance. 

**Table 2 -:** Distribution of patients according to the association of age group, sex, presence of UTI, constipation and spontaneous diuresis with UR (n=37). Uberlândia, MG, Brazil, 2020-2021

**Variables**	UR* (Yes) **n (%)**	**UR (No)** **n (%)**	**Total**	**Fisher’s exact test**
**Age group**				
Adult (< 60 years old)	8 (40.00)	12 (60.0)	20 (100.0)	0.942
Older adult (≥ 60 years old)	7 (41.18)	10 (58.82)	17 (100.0)	
Total	15 (40.54)	22 (59.46)	37 (100.0)	
**Sex**				
Female	6 (42.86)	8 (57.14)	14 (100.0)	0.823
Male	9 (39.13)	14 (60.87)	23 (100.0)	
Total	15 (40.54)	22 (59.46)	37 (100.0)	
**UTI** ^†^				
Yes	8 (66.67)	4 (33.33)	12 (100.0)	0.025
No	7 (28.00)	18 (72.00)	25 (100.0)	
Total	15 (40.54)	22 (59.46)	37 (100.0)	
**Constipation**				
Yes	15 (46.88)	17 (53.13)	32 (100.0)	0.017
No	0 (0.0)	5 (100.0)	5 (100.0)	
Total	15 (40.54)	22 (59.46)	37 (100.0)	
**Spontaneous diuresis**				
Yes	9 (75.00)	3 (25.00)	12 (100.0)	0.003
No	6 (24.00)	19 (76.00)	25 (100.0)	
Total	15 (40.54)	22 (59.46)	37 (100.0)	

^*^UR = Urinary retention; ^†^UTI = Urinary tract infection

Table 3 shows the result of the multiple binomial logistic regression analysis, adjusted for gender and age group (adult and older adult), in which patients with UTI were 7.4 times more likely to have UR when compared to patients without UTI, and this association was statistically significant (p<0.05). 

**Table 3 -:** Multiple binomial logistic regression analysis, with urinary retention as the outcome (n=37). Uberlândia, MG, Brazil, 2020-2021

**Variables**	OR* (lower limit-upper limit)	**p**
Sex	1.62 (0.32-8.04)	0.558
Age group	0.56 (0.12-2.70)	0.472
Urinary tract infection	7.40 (1.27-43.06)	0.026

^*^OR = Adjusted Odds Ratio

## Discussion

The study results showed that ICU patients were predominantly male, hospitalized for neurological causes, were sedated, used opioids during treatment, remained with the IUC for an average of 11 days, had arterial hypertension and diabetes mellitus, had UTI and intestinal constipation, during hospitalization.

The profile of this population suggests that these patients are subject to developing UR, since its frequency is higher after the age of 70, due to associated comorbidities. It is more frequent in the male population due to prostatic causes and rare in women. The length of stay of the IUC progressively increases the risk of UTI in critically ill ICU patients, hovering around 2.5% *per* day and after six days above 26.9% ^(^ 9 ^,^ 18 ^-^ 19 ^)^. 

Constipation is another triggering factor for UR, since the pathophysiology of the bladder and rectum has the same embryological origin and the same innervation, which control the function of the urinary and anal sphincters, increasing the chances of developing UR. With the reduction of intestinal motility motivated by the use of opioids analgesics, sedatives and neuromuscular blockers, intestinal constipation becomes very present in this population ^(^ 18 ^-^ 19 ^)^. 

In the assessment carried out using the RASS and Glasgow scales, the patients were drowsy, drowsy and calm, states that are very common in the ICU, often triggered by excessive use of drugs (psychotropics, opioids, analgesics and sedatives), factors those that can directly contribute to the occurrence of UR ^(3,20).^

The subjects of the present investigation spent an average of 11 days with the IUC. A study found that for each day of delay in catheter removal, the chances of infection increased by 21% and those of UR decreased by 12% ^(^ 21 ^)^. 

The use of bladder US effectively contributes to safe nursing care, as the technology brings benefits to nurses’ daily practice, cooperating with professionals in order to offer confidence in the work performed, as direct visualization of the bladder offers safety to the professional to define the right time to perform the bladder catheterization and contribute assertively to the nursing diagnosis of UR ^(^ 3 ^,^ 9 ^)^. 

Patients, when submitted to bladder US, are favored with more agile diagnoses, with a lower risk of complications, less pain and safer processes, thus reducing unnecessary catheterizations and strengthening early diagnosis ^(^ 9 ^,^ 18 ^)^. 

In this study, measurements of urinary volume by US detected up to 950 ml and a rate of 40.54% of UR. Even in the case of patients who presented spontaneous urination in a diaper, in nine of them, the measured urine volume was above 400 ml after this urination, suggesting overflow incontinence. These results reinforce the need for careful evaluations carried out by the team, as volumes above 400 ml are already compatible with urinary complaints and suggest complications such as UR ^(^ 16 ^,^ 18 ^)^. 

Research carried out in São Paulo showed that nurses considered the use of portable ultrasound to be important for greater professional autonomy, feeling safe and confident in the assessment of patients in the post-anesthesia recovery room, as performing the US facilitated the diagnosis of UR. Nurses showed concern in making a more accurate diagnosis and making immediate decisions/interventions to prevent complications ^(^ 9 ^)^. 

A study carried out in the south of Brazil found that the US examination at the bedside, performed by nurses, proved to be accurate in determining the urinary volume, and that the incidence of UR was higher when US was used for the diagnosis, when compared to patient complaint and physical examination ^(^ 3 ^)^. 

Patients with UTI were 7.4 times more likely to have UR when compared to those without UTI, corroborating the literature ^(^ 3 ^,^ 6 ^,^ 12 ^)^ which indicates early removal of the IUC, and the presence of infection can lead to other adversities, including prolonged hospitalization. 

Contrasting these results, a Japanese research found a low occurrence of UR (14.2%) in older adult men in the postoperative period of hip surgery, and the multivariate logistic regression analysis with age adjustment showed that activities of daily living (OR = 2.88) were significantly associated with the development of UR in that sample ^(^ 22 ^)^. 

It is noticed that the scenario in which the critical patient is inserted is composed of many variables. Contextualizing and reframing nursing care is essential to provide adequate nursing care, based on the best scientific parameters that result in efficiency, effectiveness and patient safety ^(^ 23 ^)^. 

Because they were sedated, the patients did not have complaints, so it was very important to carry out a physical examination and measure the urinary volume by US, after removing the IUC, which made it possible to raise the nursing diagnoses UR, risk of UR, incontinence by overflow and impaired urination and, therefore, perform a systematized and individualized nursing care for each patient.

The nursing diagnoses and interventions presented by the ICNP ^®^ Catalog were consistent with the clinical practice of intensive care nurses, making it possible to justify the conduct adopted in the face of UR outcomes, as well as the use of the clinical evaluation protocol for the diagnosis of UK nursing in adult patients ^(^ 8 ^)^, with adaptation of equipment models, for bladder scanning, available in Brazilian ICUs, without prejudice to the physical examination by the nurse. 

The limitations refer to the data collection carried out in only one hospital institution, to a small sample, which, due to the COVID-19 pandemic, had restrictions in the field and to the cross-sectional design, in which it was not possible to monitor the patients, making it difficult to the generalization of the results. Therefore, new, better-designed studies are suggested to contribute to the accuracy of the UR nursing diagnosis, using the imaging tool.

However, the research aims to contribute to health care and teaching, and especially in nursing, by presenting a theoretical framework on the use of bladder US and encouraging future research that is predisposed to test the use of the same technology in patients with UR due to through randomized clinical trials in order to support clinical practice.

## Conclusion

Thirty-seven subjects were evaluated, mostly male, whose mean urinary volume was 332.3 ml. The incidence of UR (volume measured by US above 400 ml) was statistically associated with patients with UTI, with intestinal constipation and who had spontaneous diuresis (overflow or not). Multiple binomial logistic regression showed that patients with UTI were 7.4 times more likely to have UR, when compared to those without UTI, when adjusting for sex and age group.

The results showed that 40.54% of the patients had UR, after removing the catheter, an expressive data, considering all the demand for nursing care that the critically ill patient requires, a fact that can be a complicating factor for the health of the patient.

It is suggested the implementation of bladder US in health services, as the technology brings autonomy to the professional and more safety to patients, providing better care planning, saving time and improving the quality of nursing care.
